# Supplementary figures and images for: The Dynamics of Respiratory Microbiota during Mechanical Ventilation in Patients with Pneumonia
Source: J Clin Med. 2020 Feb 27;9(3):638. doi: 10.3390/jcm9030638 (PMC7141134; doi:10.3390/jcm9030638)

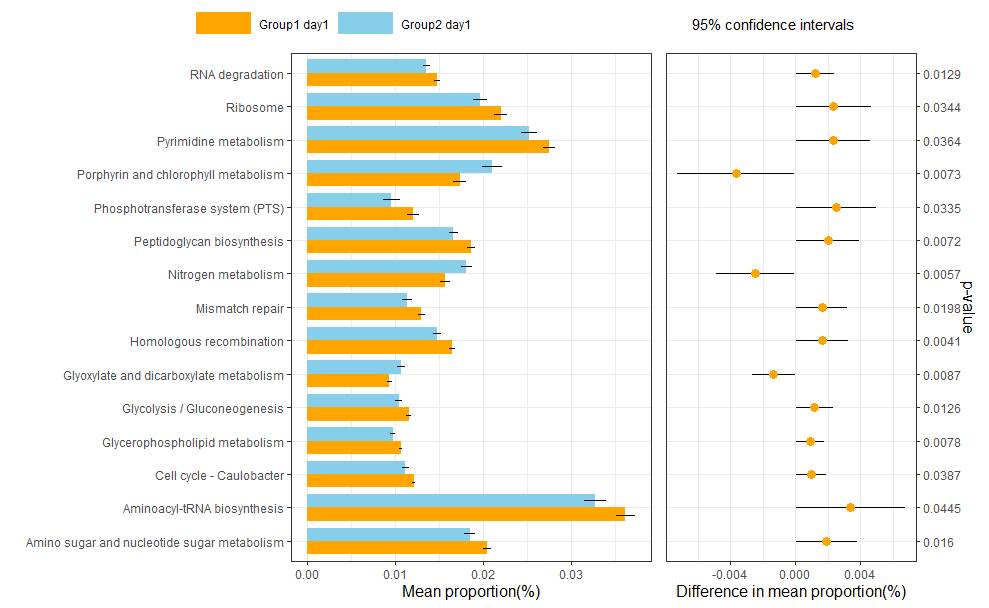

Supplement: Supplementary file 1 [file jcm-09-00638-s001.jpg]
